# Prognostic Value of NOX4 Expression in Cancer Patients: A Systematic Review and Meta-analysis

**DOI:** 10.1155/2022/8567642

**Published:** 2022-02-28

**Authors:** Hyun Min Koh, Bo Gun Jang, Chang Lim Hyun, Dong Chul Kim

**Affiliations:** ^1^Department of Pathology, Gyeongsang National University Changwon Hospital, Changwon, Republic of Korea; ^2^Department of Pathology, Jeju National University School of Medicine, Jeju, Republic of Korea; ^3^Department of Pathology, Jeju National University Hospital, Jeju, Republic of Korea; ^4^Department of Pathology, Gyeongsang National University School of Medicine, Jinju, Republic of Korea; ^5^Department of Pathology, Gyeongsang National University Hospital, Jinju, Republic of Korea; ^6^Gyeongsang Institute of Health Science, Jinju, Republic of Korea

## Abstract

**Background:**

Recent studies have shown that nicotinamide adenosine dinucleotide phosphate oxidase 4 (NOX4) is related to cancer development, proliferation, invasion, epithelial-to-mesenchymal transition, and metastasis. The prognostic value of NOX4 expression although has been reported in various cancers, it remains unclear as several studies have reported conflicting results. Therefore, the purpose of this study was to systematically investigate the prognostic value of NOX4 expression in cancer patients.

**Method:**

Appropriate studies were collected by searching the PubMed, EMBASE, and Cochrane library databases, and the prognostic value of NOX4 expression in cancer patients was assessed through a meta-analysis.

**Results:**

Nine eligible studies involving 2675 cancer patients were included in this meta-analysis. We found that NOX4 expression is related to prognosis in cancer patients. In particular, high expression of NOX4 was significantly associated with overall survival in patients with gastrointestinal cancer (hazard ratio [HR]: 1.83, 95% confidence interval [CI]: 1.39–2.42, *p* < 0.001).

**Conclusion:**

NOX4 expression is significantly correlated with overall survival in patients with gastrointestinal cancer, indicating that it could be a potential prognostic marker.

## 1. Introduction

Extensive studies over the past years suggest that reactive oxygen species (ROS) has an important role in the development and progression of cancer [1]. Nicotinamide adenosine dinucleotide phosphate (NADPH) oxidase (NOX) is one of the causes of ROS in the cells [2]. The NOX family consists of seven members, namely, NOX1 to NOX5, dual oxidase 1, and dual oxidase 2 [3, 4]. NOX members are crucial mediators of various biological mechanisms, such as cell proliferation, differentiation, apoptosis, senescence, oxygen sensing, host defense, cellular signal transduction, and inflammatory response [4]. Recent studies have revealed that NOX members are involved in the pathology of numerous diseases, including diabetes, hypertension, ischemic heart disease, atherosclerosis, fibrosis, and cancer [4].

Some cancer cells overexpress NOX members, and among them, NOX4 is often [5]. The relationship between NOX4 and cancer has been demonstrated, and enhanced ROS generated from NOX4 are known to promote cancer cell proliferation, migration, and metastasis [5, 6]. Moreover, it has been revealed that neovascularization of cancer cells is caused by NOX4 dysfunction, which induces the vascular endothelial growth factor [5–7]. Furthermore, some studies have reported the prognostic value of NOX4 in cancer, including colorectal, gastric, and endometrial cancer, hepatocellular carcinoma, esophageal and tongue squamous cell carcinoma, and retinoblastoma [5–16]. Most studies have shown that NOX4 expression is related to poor prognosis, but several studies have reported the opposite. Thus, we conducted this meta-analysis to comprehensively understand the prognostic value of NOX4 expression in cancer patients.

## 2. Methods

### 2.1. Literature Search

We collected appropriate studies through PubMed, EMBASE, and Cochrane library database searches until April 15, 2020, using the following terms: “NOX4” or “NADPH oxidase 4” and “cancer” or “tumor” or “carcinoma” or “neoplasm” or “malignancy” and “prognostic” or “predict” or “prognosis” or “survival” or “outcome.” A manual search was also performed.

### 2.2. Inclusion and Exclusion Criteria

Studies were deemed appropriate for inclusion in this meta-analysis only if the following conditions were met: (1) the association between NOX4 expression and survival was assessed; (2) the hazard ratio (HR) and 95% confidence interval (CI) for survival were presented; and (3) NOX4 expression was investigated in human cancer cells. The following studies were excluded from the analysis: (1) reviews, case reports, letters, conference abstracts, and non-English articles; and (2) duplicate studies.

### 2.3. Data Extraction

We collected the following information from the included studies: First author, publication year, country, cancer type, sample size, sex, mean or median age of the patients, study period, follow-up period, NOX4 expression associated with poor prognosis, NOX4 expression cut-off value, and HR with 95% CI for survival. The data were independently extracted by two authors, and any conflicts or difference in opinions were resolved consensually.

### 2.4. Quality Assessment

We used the Newcastle-Ottawa Scale to assess the quality of the included studies. Quality reviews were independently conducted by two authors, and any differences in the evaluation results were resolved through a consensus.

### 2.5. Statistical Analyses

Meta-analysis was performed to calculate the effect size among the included studies. I^2^ was used to evaluate the heterogeneity between each study. Subgroup analysis was performed to determine the cause of heterogeneity. Funnel plots and Egger's tests were used to check for publication bias, and sensitivity analysis was conducted as a consistency evaluation of the pooled results. A *p* value < 0.05 was considered statistically significant. All analyses were performed using Stata/SE17 (Stata, College Station, TX, USA).

## 3. Results

### 3.1. Characteristics of the Included Studies

**Figure 1:**
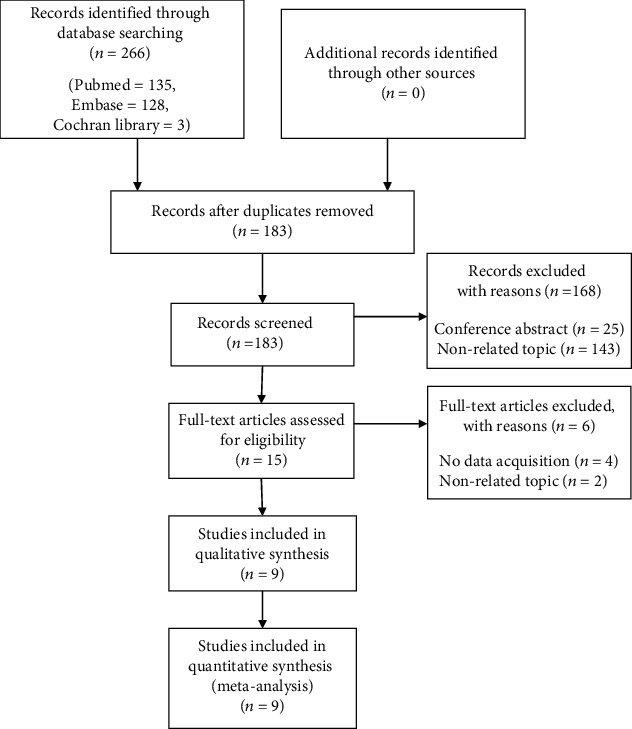
Flow diagram of study collection.

We selected nine eligible studies through a review of the literature (Figure 1). The basic information regarding the selected studies is summarized in Table 1. The publication year of the studies ranged from 2016 to 2019, and all other studies except one were published in Asia. The cancer types included were hepatocellular carcinoma (*n* = 3), colorectal cancer (*n* = 2), gastric cancer (*n* = 1), esophageal squamous cell carcinoma (*n* = 1), tongue squamous cell carcinoma (*n* = 1), and endometrial cancer (*n* = 1). The sample size of all included studies was between 82 and 876, with a total of 2675 cancer patients. Immunohistochemistry was most commonly used method to detect NOX4 expression; other methods such as analyses of The Cancer Genome Atlas, Human Protein Atlas, and Oncomine data as well as reverse transcription-polymerase chain reaction were also used. Regarding the association of NOX4 expression with poor prognosis, seven studies reported that high NOX4 expression was related to poor prognosis, whereas the other two studies reported low NOX4 expression was related to poor prognosis. The qualitative assessment scores of the included studies were rated relatively good, ranging from 6 to 8.

### 3.2. Association between NOX4 Expression and Overall Survival

**Figure 2:**
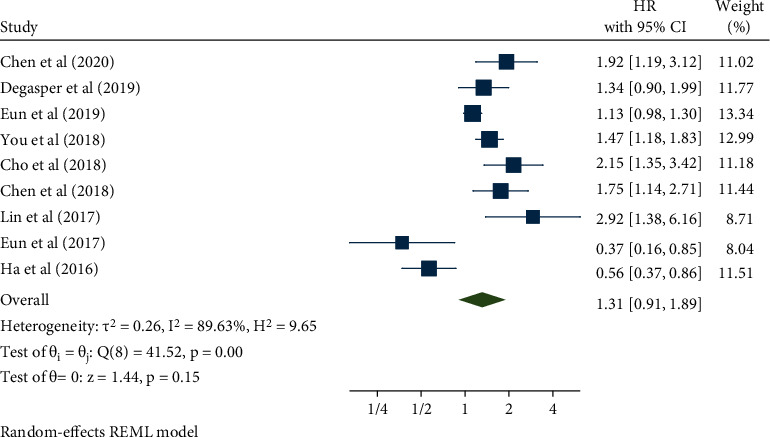
Forest plot of the association between NOX4 expression and overall survival.

**Figure 3:**
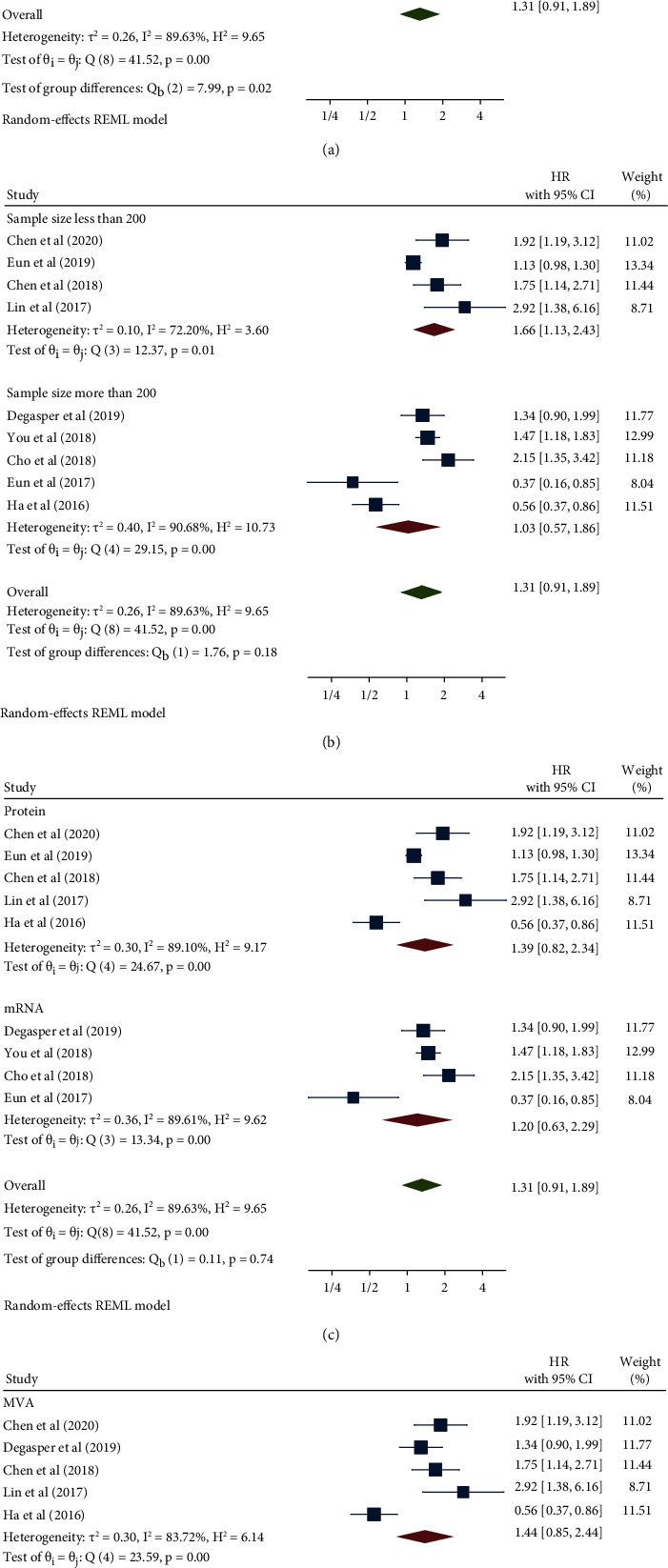
Forest plot of the association between NOX4 expression and overall survival stratified by cancer type (a), sample size (b), NOX4 detection method (c), and survival analysis (d).

The nine studies included in this analysis, which involved a total of 2675 cancer patients, revealed an association between NOX4 expression and overall survival (OS). The pooled HR with 95% CI was demonstrated using a meta-analysis with a random-effects model (*I*^2^ = 89.63%, *p* < 0.001). The results implied an association between high expression of NOX4 and poor OS in cancer patients (HR: 1.31, 95% CI: 0.91–1.89, *p* = 0.15) (Figure 2). To find the sources of heterogeneity, subgroup analysis was performed (Table 2). With respect to the cancer type, the result was consistent with the pooled results in the gastrointestinal type (HR: 1.83, 95% CI: 1.39–2.42, *p* < 0.001) and in other cancers (HR: 1.51, 95% CI: 1.13–2.03, *p* = 0.01), but not in hepatocellular carcinoma (Figure 3(a)). The result was also compatible with the pooled results in a sample size of less than 200 patients (HR: 1.66, 95% CI: 1.13–2.43, p =0.01), but not in more than 200 (Figure 3(b)). Considering NOX4 detection and survival analysis, not all subgroups showed meaningful results (Figures 3(c) and 3(d)). In addition, meta-regression was implemented, but no statistically significant results were obtained for any of the subgroups.

### 3.3. Association between NOX4 Expression and Disease-Free Survival

**Figure 4:**
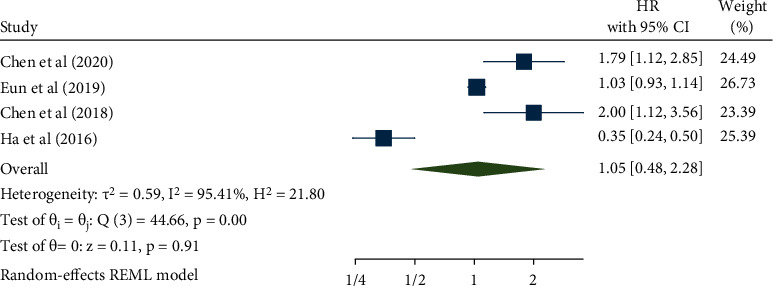
Forest plot of the association between NOX4 expression and disease-free survival.

**Figure 5:**
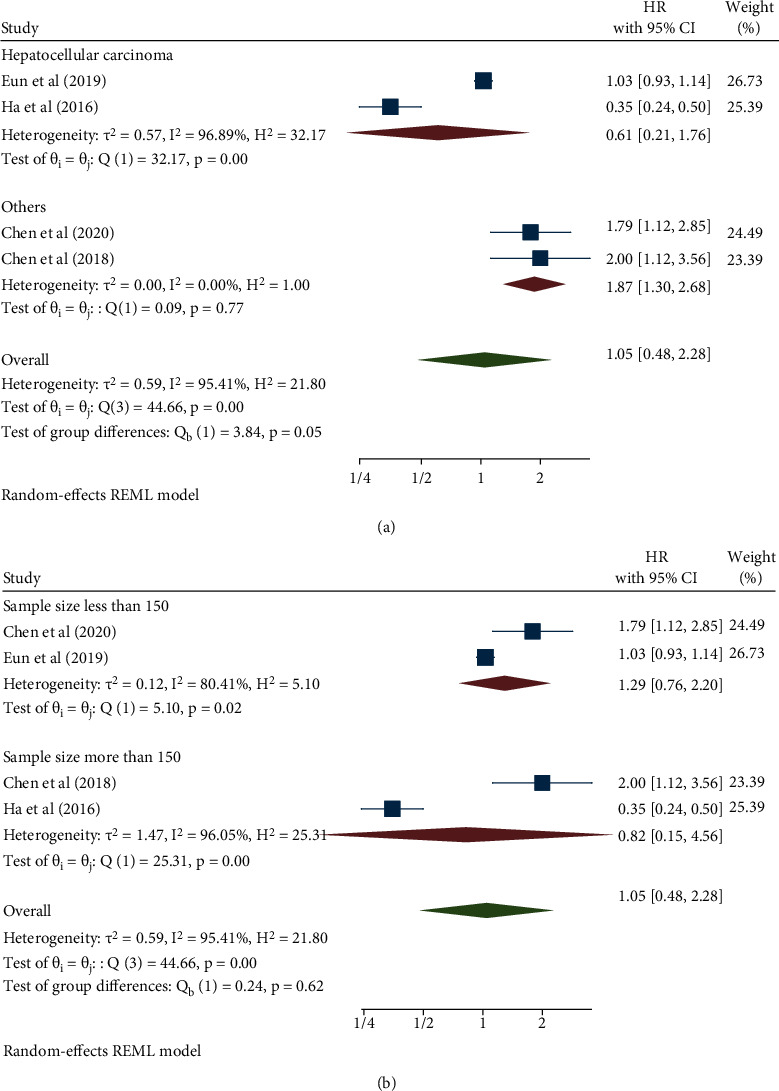
Forest plot of the association between NOX4 expression and disease-free survival stratified by cancer type (a) and sample size (b).

Four studies with a total of 643 cancer patients revealed an association between NOX4 expression and disease-free survival (DFS) and recurrence-free survival (RFS). In this study, RFS was included in the DFS and was analyzed using the random-effects model (*I*^2^ = 95.41%, *p* < 0.001). The pooled HR was 1.05 (95% CI: 0.48–2.28, *p* = 0.91) (Figure 4). Nevertheless, although the results suggested an association between NOX4 expression and DFS in cancer patients, it was not statistically significant. Additionally, we performed a subgroup analysis on cancer type and sample size (Table 3). With regard to the cancer type, the results indicated an association between high NOX4 expression and poor DFS in the subgroup of all other cancers (HR: 1.87, 95% CI: 1.30–2.68, *p* < 0.001), but not with hepatocellular carcinoma (Figure 5(a)). For the sample size, no subgroup obtained statistically significant results (Figure 5(b)).

### 3.4. Publication Bias

**Figure 6:**
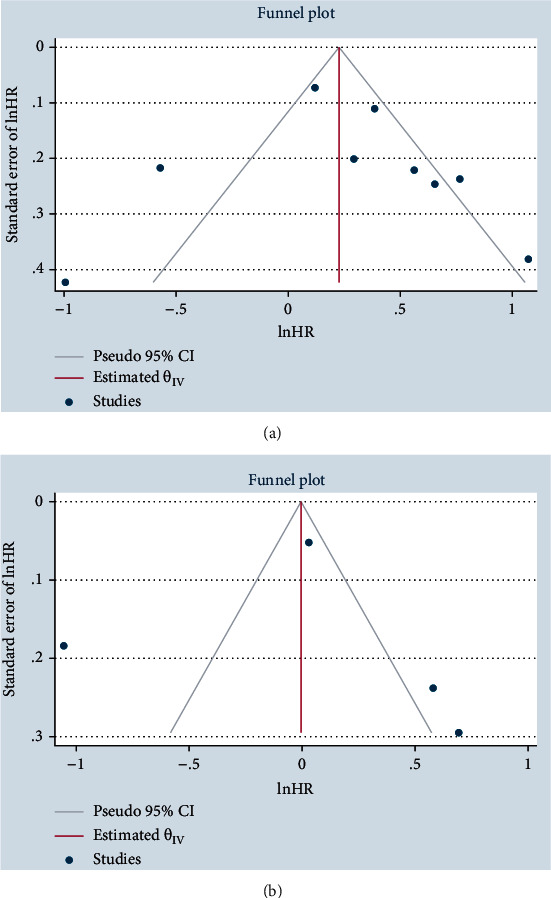
Funnel plot of the association between NOX4 expression and overall survival (a) or disease-free survival (b).

We constructed a funnel plot and performed Egger's test to check for publication bias in terms of OS and DFS (Figures 6(a) and 6(b)). However, Egger's test was not significant for OS (*p* = 0.76) and DFS (*p* = 0.55), demonstrating no small-study effects.

### 3.5. Sensitivity Analysis

**Figure 7:**
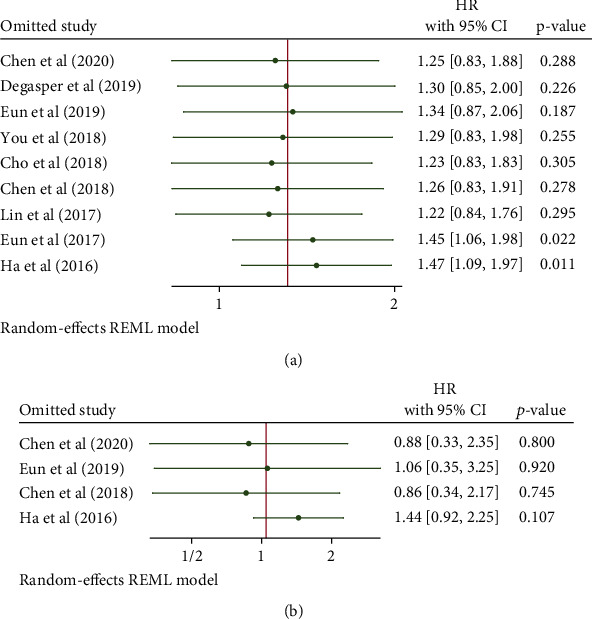
Sensitivity analysis of the association between NOX4 expression and overall survival (a) or disease-free survival (b).

We conducted a sensitivity analysis to examine the effects of individual studies. The results revealed that individual studies had an influence. Nevertheless, it did not have an impact on the overall results for OS (HR: 1.31, 95% CI: 0.91–1.89, *p* = 0.15) and DFS (HR: 1.05, 95% CI: 0.48–2.28, *p* = 0.91) (Figures 7(a) and 7(b)).

## 4. Discussion

The NOX family are enzymes that have the ability to generate superoxide or hydrogen peroxide, which is one of the major endogenous sources of ROS [17, 18]. NOX4, a member of the NOX family, is abundantly expressed in human tissue, especially in blood vessels and the kidney [19]. Physiologically, NOX4 is involved in various cellular responses through ROS generation [19]. Low levels of ROS can contribute to the signal transmission needed for cell proliferation, differentiation, migration, apoptosis, and oxygen sensing; by contrast, high levels of ROS can cause cell damage or death [19]. Many recent studies have shown that NOX4 and generated ROS are related to cancer development, proliferation, invasion, epithelial-to-mesenchymal transition, and metastasis [19–22]. Moreover, the prognostic value of NOX4 expression has been reported as numerous cancers, such as colorectal, gastric, and endometrial cancer, hepatocellular carcinoma, esophageal and tongue squamous cell carcinoma, and retinoblastoma [5–16]. However, there has been no systematic analysis of the relationship between NOX4 expression and prognosis in cancer patients.

In this study, we first evaluated the prognostic and clinicopathological value of NOX4 expression in cancer patients. We collected nine studies that included a total of 2675 cancer patients and conducted a meta-analysis using data extracted from these studies. Our results suggested that NOX4 expression was related to OS and DFS in cancer patients, but this association was not statistically significant. However, through subgroup analyses, we also demonstrated that NOX4 expression is significantly associated with poor OS in gastrointestinal and other cancers and with poor DFS in other cancers. We further revealed that NOX4 expression is significantly correlated with poor OS in the subgroup with a sample size of less than 200 patients.

Most of the studies we collected reported that high expression of NOX4 was related to poor prognosis. In contrast, two studies demonstrated that low expression of NOX4 was associated with poor prognosis; interestingly, both of these studies (Eun et al. [10] and Ha et al. [12]) were conducted on hepatocellular carcinoma, whereas another study (Eun et al. [11]) on hepatocellular carcinoma also revealed the opposite result. We believe that these conflicting findings could have a significant impact on the overall results of this meta-analysis. Thus, we suggest that further studies should be conducted on the impact of NOX4 expression in hepatocellular carcinoma.

Despite various efforts, our research has several limitations. First, all of the studies we collected, except for one, were published in Asia. Therefore, the applicability of our results to other regions is questionable. Second, the heterogeneity of the included studies was high because of diverse detection methods, cut-off values, and survival analysis of NOX4 expression. We recommend the collection of articles using a relatively consistent study method and performing another meta-analysis if further research becomes available in the future.

Nevertheless, in the present study, we reported a systematic review of the relationship between NOX4 expression and prognosis in cancer patients. In summary, NOX4 expression was significantly related to prognosis in patients with gastrointestinal cancer, indicating that NOX4 expression might be a potential prognostic marker for gastrointestinal cancer.

## Figures and Tables

**Table 1:** Basic information of the selected studies.

Study	Country	Cancer type	Sample size	Sex (male/female)	Mean or median age (years)	Study period	Follow-up (months)	Survival outcome	NOX4 detection method	NOX4 expression associated with poor prognosis	Cut-off value of NOX4 expression	Survival analysis	NOS
Chen et al. (2020) [5]	Taiwan	Esophageal squamous cell carcinoma	121	116/5	Mean55 (29-80)	2001-2012	NA	OS, DFS	IHC	High expression	≥50%	MVA	7
Degasper et al. (2019) [6]	Austria	Endometrial cancer	239	-	Median 69 (37-93)	1989-2015	NA	OS	RT-PCR	High expression	>76 percentile	MVA	7
Eun et al. (2019) [11]	South Korea	Hepatocellular carcinoma	134	103/31	Mean58.1	1999-2014	Mean 49.15 (OS), 34.92 (DFS)	OS, DFS	IHC	High expression (Nuclear)	Staining scores with intensity and proportion (>3)	UVA	7
You et al. (2018) [16]	China	Gastric cancer	876	NA	NA	NA	NA	OS	HPA and Oncomine data	High expression	>median expression	UVA	6
Cho et al. (2018) [7]	South Korea	Colorectal cancer	458	242/216	NA	NA	NA	OS	TCGA data	High expression	>39.13	UVA	6
Chen et al. (2018) [8]	Taiwan	Tongue squamous cell carcinoma	161	148/13	Median 53 (26-86)	2006-2015	62.8 (2.3-117.6)	OS, DFS	IHC	High expression	≥50%	MVA	8
Lin et al. (2017) [13]	China	Colorectal cancer	82	47/35	NA	2009	1-77	OS	IHC	High expression	Staining scores with intensity and proportion (≥4)	MVA	8
Eun et al. (2017) [10]	South Korea	Hepatocellular carcinoma	377	255/122	NA	NA	NA	OS	TCGA data	Low expression	<43.96	UVA	6
Ha et al. (2016) [12]	South Korea	Hepatocellular carcinoma	227	135/92	Median 53	2000-2006	120.3 (14-151.4)	OS, RFS	IHC	Low expression	≤50%	MVA	8

DFS, disease-free survival; HPA, Human Protein Atlas; IHC, immunohistochemistry; MVA, multivariate analysis; NA, not available; NOS, Newcastle-Ottawa Scale; NOX4, NADPH oxidase 4; OS, overall survival; RFS, recurrence-free survival; RT-PCR, reverse transcription-polymerase chain reaction; TCGA, The Cancer Genome Atlas; UVA, univariate analysis.

**Table 2:** Subgroup analysis and meta-regression of the association between NOX4 expression and overall survival in cancer patients.

Subgroup					Heterogeneity		Meta-regression
	Number of studies	Number of patients	Pooled HR (95% CI)	p value	I^2^ (%)	p value	p value
Cancer type							0.09
GI cancer	4	1537	1.83 (1.39-2.42)	< 0.001	40.90	0.18	
HCC	3	738	0.67 (0.36-1.27)	0.23	85.89	< 0.001	
Others	2	400	1.51 (1.13-2.03)	0.01	0.00	0.37	
Sample size							0.23
Less than 200	4	498	1.66 (1.13-2.43)	0.01	72.20	0.01	
More than 200	5	2177	1.03 (0.57-1.86)	0.86	90.7	< 0.001	
NOX4 detection							0.77
mRNA	4	1950	1.20 (0.63-2.29)	0.29	89.61	< 0.001	
Protein	5	725	1.39 (0.82-2.34)	0.16	89.10	< 0.001	
Survival analysis							0.62
MVA	5	830	1.44 (0.85-2.44)	0.16	83.72	< 0.001	
UVA	4	1845	1.15 (0.62-2.15)	0.26	94.59	< 0.001	

CI, confidence interval; GI, gastrointestinal; HCC, hepatocellular carcinoma; HR, hazard ratio; MVA, multivariate analysis; NOX4, NADPH oxidase 4; UVA, univariate analysis.

**Table 3:** Subgroup analysis and meta-regression of the association between NOX4 expression and disease-free survival in cancer patients.

Subgroup					Heterogeneity		Meta-regression
	Number of studies	Number of patients	Pooled HR (95% CI)	p value	I^2^ (%)	p value	p value
Cancer type							-
HCC	2	361	0.61 (0.21-1.76)	0.36	96.89	< 0.001	
Others	2	282	1.87 (1.30-2.68)	< 0.001	0.00	0.77	
Sample size							-
Less than 150	2	255	1.29 (0.76-2.20)	0.35	80.41	0.02	
More than 150	2	388	0.82 (0.15-4.56)	0.82	96.05	< 0.001	

CI, confidence interval; HCC, hepatocellular carcinoma; HR, hazard ratio; NOX4, NADPH oxidase 4.
